# Understanding the Experiences and Needs of Migrant Women Affected by Female Genital Mutilation Using Maternity Services in Australia

**DOI:** 10.3390/ijerph17051491

**Published:** 2020-02-26

**Authors:** Sabera Turkmani, Caroline S. E. Homer, Angela J. Dawson

**Affiliations:** 1 Australian Centre for Public and Population Health Research, Faculty of Health, University of Technology Sydney, Ultimo NSW 2007, Australia; angela.dawson@uts.edu.au; 2 Maternal, Child and Adolescent Health Program, Burnet Institute, Melbourne VIC 3004, Australia; caroline.homer@burnet.edu.au

**Keywords:** female genital mutilation (FGM), women’s health needs, equality, quality of maternity care, midwifery continuity of care

## Abstract

Female genital mutilation (FGM) is a cultural practice defined as the partial or total removal of the external female genitalia for non-therapeutic reasons. Changing patterns of migration in Australia and other high-income countries has meant that maternity care providers and health systems are caring for more pregnant women affected by this practice. The aim of the study was to identify strategies to inform culturally safe and quality woman-centred maternity care for women affected by FGM who have migrated to Australia. An Appreciative Inquiry approach was used to engage women with FGM. We conducted 23 semi-structured interviews and three focus group discussions. There were four themes identified: (1) appreciating the best in their experiences; (2) achieving their dreams; (3) planning together; and (4) acting, modifying, improving and sustaining. Women could articulate their health and cultural needs, but they were not engaged in all aspects of their maternity care or considered active partners. Partnering and involving women in the design and delivery of their maternity care would improve quality care. A conceptual model, underpinned by women’s cultural values and physical, emotional needs, is presented as a framework to guide maternity services.

## 1. Introduction

Female genital mutilation (FGM) is defined as the partial or total removal of external female genitalia for non-therapeutic reasons [1]. This practice is deeply rooted in culture, with social or religious obligation and marriageability considered to be the most important reason for its continuation (UNICEF 2013). FGM is also performed for fear of being excluded from opportunities as a young woman [2]. FGM is traditionally practiced in 30 African and Middle Eastern countries, and some parts of Asia and South America [3]. Changing patterns of migration have led to an increase in the prevalence of women with FGM in many high-income countries [4,5].

It is estimated that globally over 200 million women and girls have undergone FGM and another three million women and girls are at risk annually [6]. There is a lack of reliable and high-quality data in relation to the numbers of women affected by FGM in high-income countries (HICs) [7]. It is, therefore, challenging for countries to develop effective policies, allocate relevant resources and evaluate the results of interventions [8,9]. In Australia, a recent report [10] estimated that in 2017 there were 53,000 women and girls affected by FGM in Australia, which represents a prevalence of 4 per 1000 girls and women. 

Women affected by FGM in HICs are usually migrants or refugees and may have complex needs in addition to their clinical care [11,12]. These women are more likely to face socio-economic and cultural challenges due to language barriers, low education levels, and financial difficulties, which can hinder access to health services [13,14]. Migrant and refugee women from low- and middle-income countries, especially those from African countries, are reported to have poorer perinatal outcomes due to a higher rate of complications during pregnancy and childbirth [15,16]. FGM poses an additional burden to affected women and there are potential adverse consequences during pregnancy and childbirth such as an increased risk of caesarean section, post-partum haemorrhage, instrumental birth and prolonged labour [6]. 

Research has found that health services in some HICs may not be adequately prepared to provide quality care to FGM-affected women [17]. For example, many health professionals lack clinical skills and knowledge about the law in relation to FGM [17,18]. Health professionals have also been found to have a poor understanding of the cultural background of women affected by FGM and find communication challenging [19,20]. These issues, combined with inadequate support services, such as interpreting and counselling services, mean that many women may face difficulties expressing their needs [21]. 

The World Health Organization (WHO) highlights the importance of improving the quality of maternity care for women with complex needs to minimise further complications and harm [6]. The WHO’s quality care standards outline eight domains of quality of care that encompass the provision of care and a woman’s experience of care [22]. Quality of health services would improve if women trust and are confident to utilise the services on the basis of their positive and satisfactory experiences [23]. While research has provided insight into what constitutes quality maternal care from a health system perspective, gaps remain concerning the views and needs of women with FGM and what they regard as quality care. This study aimed to identify approaches to achieve culturally safe and high-quality woman-centred care for migrant women who have been personally affected by FGM. 

## 2. Materials and Methods

The study employed Appreciative Inquiry (AI), a qualitative methodology to gain a deep insight into women’s experiences of midwifery care in an Australian setting [24]. Appreciative Inquiry is a well-accepted methodology in health research [25]. This methodology has been used to explore patient experience of clinical healthcare [26] and to address the complex needs of families and children in primary health care [27]. McAdam and Mirza [28] used AI to describe the experience of marginalised youth engaged in drug and alcohol misuse and the implications of positive stories on health and social well-being.

We applied the four-stepped processes of AI to elicit examples of positive care interactions and envisage what best quality maternity care might look like in the future [29]. AI is open-ended, allowing a flexible approach to be taken depending on the needs of the participants [30]. The collaborative nature of AI is helpful in the development of confidence and can motivate participants to become actively involved in change [31]. We designed this research not only to identify the changes that are needed to improve maternity care but encourage women to become involved in this change as users and beneficiaries of maternity services.

Ethical considerations for this project were of particular importance as the migrant women with FGM often feel vulnerable, stigmatised and marginalised [32]. Ethical approval (UTS HREC REF NO. ETH17-1525) was obtained from the Human Research Ethics Committee of UTS in August 2017 before the recruitment or data collection process.

This study was conducted in Western Sydney, New South Wales (NSW), the area that has the largest number of non-English speaking women in this state of Australia [33]. The participants were English-speaking migrant and refugee women who were personally affected by FGM and lived in Sydney. The women had given birth in Australia in the last ten years or were currently pregnant. A written information package was given to women to invite them to participate. The research was designed and conducted in direct consultation with experts in this area from government and non-governmental organizations, and an independent activist and advocate who is a survivor of FGM. In addition, a member of the community was involved throughout the study process and guided the development of the research tools, assisted with recruitment of the study population and ensured that the project was conducted in an ethical and culturally appropriate manner. Chain referral sampling was employed to approach potential women [34]. This method of sampling is useful for recruiting participants in research where the topic is sensitive or in populations that are stigmatised and hard to reach [35]. Participants signed the informed consent form prior to the commencement of interviews or group discussions. Participant anonymity was assured by allocating a code to the woman’s name. 

Data were gathered through in-depth interviews and followed by focus group discussions over five months (October 2017–February 2018). Interviews lasted for one hour and group discussions were conducted with five to eight women for two hours. The interviews and discussions were guided by questions (Supplementary Materials) following concepts of AI that were flexible enough to enable an exploration of ideas and experiences that women raised [36]. Interviews were held at a time and place convenient to women and group discussions were held in community centres. We offered small gift cards as compensation for their time and travel to the interview location. 

Braun and Clarke’s [37] approach to qualitative data analysis was followed because it offered a way of analysing the data according to the 4Ds representing the discovery, dream, design and deploy phases of AI in the first instance followed by a closer analysis of women’s experiences as maternity service users.

The interview transcripts were first transcribed verbatim [38] enabling the researchers to familiarise themselves with the data tomake sense of it and reflect on overall meanings and general ideas [39]. Data were exported into the NVivo qualitative data management software to enable coding of the text according to the four phases of AI. Woman’s narratives were coded into themes representing 4Ds and appropriate sub-themes [40]. The final step of data analysis involved the interpretation of data to draw recommendations for future maternity care policy and practice [39]. 

## 3. Results

In total, 23 individual interviews and three focus group discussions were conducted. The women were from Sudan (*n* = 9), Somalia (*n* = 6), Sierra Leone (*n* = 3), Egypt (*n* = 2), Indonesia (*n* = 2), and Ethiopia (*n* = 1). The majority of women (*n* = 21) had undergone FGM when they were 0-10 years old. All of the women came to Australia as refugees except for four who entered the country on spousal visas or employment visas. English was the second language for all women (Table 1).

**Figure 1:**
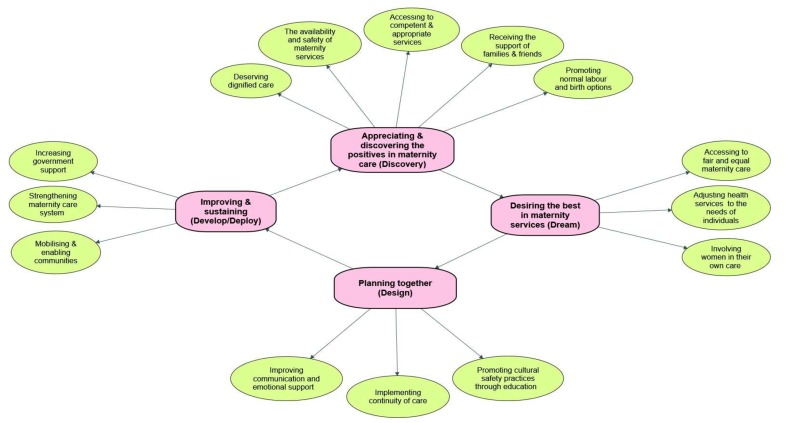
Thematic data analysis based on 4Ds cycle (stages) of Appreciative Inquiry.

The main findings are presented under four themes in line with the 4D cycle of Appreciative Inquiry: Appreciating the positives in their maternity care (Discovering); Desiring the best in maternity services (Dreaming); Planning together for improved maternity services (Designing); Improving and sustaining (Developing/Deploying). The four themes and their associated sub-themes are further elaborated in the sections below (Figure 1).

### 3.1. Appreciating and Discovering the Positives in Maternity Care (Discovering)

Appreciating the positives in maternity care concentrated on women’s description of events during their maternity care in Australia, and the strategies or approaches that they perceived to be useful, or inappropriate. For the most part, women were appreciative of, and satisfied with, the maternity care they received. This included being provided with respectful care, a feeling of having a safe service, receiving the required information, having access to skilled health care providers, and being able to have advance care planning and family support. Women frequently reported that “Maternity services are really good in Australia compared to where we came from”.

Women felt that the maternity services are safe and technologically advanced in Australia and they expected their maternity care providers to have an appropriate level of knowledge about FGM, possess effective communication skills, be sensitive to their cultural needs and involve women in their care. For example:

*The good thing was always feeling safe, knowing there are all the facilities, medicines and machines and skills you might need available within the hospital. I really felt relaxed in both my deliveries. Overall pregnancy was a happy experience for me and I knew they would help me straightaway compared to my country where nothing is available.*

(W18)

Most of the women appreciated maternity care providers who were helpful, sensitive and responsive to their needs, especially when they had no family and relatives around to support them. Women were impressed by the way maternity care providers made them feel cared for, particularly when they followed up to make sure women did not miss their appointments. 

A few women thought that while caring for women with FGM was not a common experience for Australian maternity care providers, women expected providers to know how to deal with FGM and how to communicate with women. This woman said:

*… it is not like that the doctors and midwives in Australia come across a circumcised woman every day, you know. And I don’t blame them if they are surprised or ask you millions of questions.*

(W13)

Several women were worried about the care they might receive because they did not think maternity care providers were adequately prepared to manage FGM as this woman explained:

*The medical staff need to understand this issue [FGM] and be knowledgeable about it and if they don’t have hands-on experience and skills please do not touch us and make our situation worse. You need to feel safe knowing that they get training before coming to women with FGM.*

(W23)

The women acknowledged that developing trust with a maternity care provider was directly associated with the provider’s competence. Some felt anxious and lost confidence in the ability of maternity care providers to deliver good care when they saw that their care providers were surprised or shocked when they encountered FGM. One woman in a focus group said:

*If these midwives and doctors know where to cut (de-infibulation), how to cut and when to cut it will be so helpful for us and for them because we will not have a problem and they will be relaxed and confident in what they do. Now, as soon as they see us they are shaking … Oh my God. They can get advice from doctors and midwives who worked in our country and have real experience of treatment of women with FGM.*

(FGD3)

### 3.2. Desiring the Best in Maternity Services (Dreaming)

Women expressed their vision for the best maternity care in the future, including how they would wish to be treated within the healthcare system. They described the need for equality and for FGM-affected women to be treated the same way as other women. This included a desire for personalised care to be delivered by a provider from a similar cultural background with services tailored to the needs of the individual woman. In practical terms, women described how individualised care should mean the provision of support services for women following de-infibulation. 

Women believed that each pregnancy is an individual experience and expressed a clear understanding of the need for services to facilitate informed choice and shared decision making in a way that involves women with FGM in their own care. They wanted maternity care providers to listen to each woman and adjust care to suit her individual needs, rather than following the same course for every woman. One woman said:

*They need to listen to women as they know their body better. Not everything is going to be according to the recipe in the book. They have to look at each individual pregnancy separately.*

Women wanted to be treated in the same way as other women without being labelled as different while accessing Australian maternity services. They also wanted to have access to appropriate mental health support that took into consideration their special circumstances due to their FGM, for example:

*If a woman has undergone FGM they need to look after her even after birth and even if there is not any visible harm there is always a change and she needs that emotional support.*

(W20)

Many women struggled with their body image and the emotional impact of de-infibulation, and some wished to see their bodies the way they were used to seeing it since childhood (infibulated). This was exacerbated by the fact that legislation in the state did not provide the option of re-infibulation. They perceived a reluctance of health staff to consider any form of reconstruction of the vulval or perineal area and attributed this to laws prohibiting re-infibulation. Women wanted reconstructive surgery to be part of the services offered to them. Most believed that their de-infibulation had been done ‘badly’ and their body would be ‘in better shape’ if they were re-infibulated after birth. They desired varying degrees of re-infibulation and used the term ‘closed-back’ when describing reconstructive surgery. This comment captures such feelings:

*After they open you during delivery I wish there is someone who stitches it very very nicely so it doesn’t look very open.*

(FGD1)

Most women felt embarrassed and uncomfortable with their bodies and described their vulva as ‘ugly’, ‘too open’, ‘not in good shape’, ‘hanging skin’, and ‘horrible’. Some women chose to undergo a caesarean section to avoid de-infibulation or they travelled back to their home country to be re-infibulated as this woman did:

*…I went overseas and closed it by a midwife in my country. You know last time I [got] closed myself in Sudan it was because it was so big and ugly they left me totally open at least they could have stitched me back to make me look like normal.*

(W22)

Women recognised and valued their capacity to experience birth as a normal process without unnecessary intervention. Some wished that their FGM was not considered as a barrier to undergoing normal labour and birth and questioned interventions such as caesarean section. Several women stated, ‘We had our baby normally and easier in our country; why not here’. 

Some women felt they were vulnerable, disempowered and dominated by maternity care providers and these providers took control of the situation. Some agreed to let their family members make decisions on their behalf, while others expressed their strong desire to be involved in a collaborative way with maternity care providers. Culture, personal attitudes, and emergencies were also identified by most women as factors influencing the degree to which they could be involved in decision making. For example:

*My husband and mother in law made the decision for me. If it was up to me I would have chosen a caesar straightaway. I did not want all that pain and trauma, but midwife went with my husband and mother in law’s decision without listening to me.*

(FGD1)

Many women perceived that their lack of health literacy and knowledge about access to certain options or health services led to their exclusion from decision making. One woman explained:

*Sometimes you are in a position where you have to follow whatever they say. Maybe because our knowledge is limited and the language also is a big, big problem.*

(W18)

### 3.3. Planning Together for Improved Maternity Services (Designing)

The ‘design’ phase of AI invited women, individually or as part of a group, to develop a plan for what they need to achieve their dream for quality care. 

In designing future maternity services, women discussed the need for education initiatives that enabled maternity care providers to provide emotional support, promote cultural safety and communicate in ways that are appropriate for supporting women with FGM. The need for training to involve women themselves to improve provider understanding of and familiarity with the cultural beliefs behind the practice of FGM was highlighted, as these women explained:

*If I am a midwife I make you feel good and I need to understand what you believe in so I can understand if you see FGM as a good thing or bad thing. Then I can talk to you and guide you accordingly… first you need to get a sense of what women believe in, otherwise they may not disclose anything.*

(W12)

Women also noted that, while maternity care providers need to be respectful and integrate the cultural aspects into their services, they also need to be mindful of harmful cultural practices that may place women at risk. For example, in some cultures, women do not use direct communication to explain their problems related to childbirth, maternity care or FGM. Many women mentioned that they avoided disclosing their FGM as they thought this was culturally inappropriate, as explained here:

*I was shy and hide my FGM until birth and I am sure many other would do that. In our culture women won’t talk about it believe me or not. There is shame and stigma with those topics’.*

(W23)

Midwifery continuity of care was one of the models of care or services most appreciated by women who received it. Women understood midwifery continuity of care as being cared for by a known midwife over the entire period of pregnancy and childbirth and after birth. Being with the same midwife and building a relationship based on mutual trust and understanding was perceived to improve women’s sense of safety and confidence and increase their involvement in their care. Most women, however, did not have access to this model of care. There were a few women who received midwifery continuity of care during pregnancy, but during labour and birth, their known midwives were not present. They expressed feelings of anxiety and distress with being cared for during labour and birth by midwives they had not met before. Women suggested that maternity services should be designed to enable all women to have access to such a model of care, for example:

*It is very important for women because we want to trust someone and by changing midwives and doctors we will be lost. I will also develop my confidence in her competence and make sure she can manage my birth and I am in safe hands. That’s a huge support for me knowing that I am safe and someone knows my issues and concerns.*

(W17)

Women viewed high-quality maternity care in terms of the way that maternity care providers had behaved towards them. Considering the sensitivity of a topic such as FGM, the women believed positive and effective communication was a key component of maintaining a sense of connection, trust, and collaboration with health providers. For example, they wanted to be heard, touched and welcomed. Many women indicated that building trust happened over time as they got to know their maternity care providers through their direct interactions. As this woman explains, this was especially important in addressing the embarrassment that many women felt because of their FGM:

*You know little by little each time after I started to visit the doctors and midwives and they didn’t make me feel embarrassed [because of FGM] and they asked me so many questions when I went to them. And the way they talked to me was so good. You know, you feel so good when someone listens to you. They were not in a rush to get to the next patient and kick me out of their office. They spend time with you and do what they need to do while they kept privacy.*

(W13)

Women wanted to receive emotional support to address their trauma including dealing with health issues related to FGM.

*Sometimes you just want someone to talk to and ask for nothing else, just someone to ask you what your feelings after birth are or how you are because it is a hard time. … I want a midwife or nurse to provide care for me beyond giving medicines, I want them to talk to me and support me emotionally and mentally.*

(W17)

Many women felt that there was no transparent, clear, and mutual communication between them and their maternity care providers. As a result, women were often suspicious of the maternity care services they received and were not always willing to accept advice from maternity providers as illustrated with the following quote:

*Sometimes they don’t even talk about FGM with us and just write everything down and say all is good without giving us the details. I think it is mostly because they don’t know anything about FGM and they just look at you and they have no idea.*

(W17)

### 3.4. Improving and Sustaining Maternity Services (Developing/Deploying)

The final theme reflected strategies that women regarded as useful to support their plans to improve maternity services. The women’s suggestions represented three levels of action: mobilising and enabling communities, strengthening maternity care systems and increasing government support. 

Women believed that communities need to be mobilised to create a supportive environment in which pregnant women and new mothers affected by FGM can feel safe and healthy. Advocacy and campaigns for policy, professional practice, and at a community level were considered critical in creating a supportive environment to improve health outcomes for women in the long term. Raising community awareness, through formal and informal education, campaigns in the community and schools, and involving women, men and young people, were considered essential to delivering positive change. One woman said:

*Still many people in the community believe it is a good thing to do on their daughters [FGM]. … I will not let my daughter to undergo FGM but we need to remove pressure of the community on families. If no one wants a girl without FGM then everybody forced to do it. We need to end that by educating community and change this culture.*

(W22)

Women believed the practice of FGM was continuing in their communities, even in Australia, and emphasised the need for a reporting system at the community level. Women stated that the success of community-based interventions, such as education and media campaigns, depends upon the involvement of all members of the community including religious and community leaders in the planning and implementation processes of change. Women emphasised the central role of families in bringing a sustainable change to stop a culture such as FGM as explained here:

*Change is dependent on families. In my family, I have already talked to my kids about the stuff like FGM and the even bigger impact of it on society. I think that’s how we will spread the word and stop it, otherwise it is never going to be stopped. Now people believe in this society that talking about this issue is wrong or Haram [prohibited by religion]. I don’t care; I will talk to my children because I don’t want them to grow up blindly.*

(W18)

Men were regarded as important actors of change, but women thought that they lacked knowledge about the physical and mental health consequence of FGM. Women felt that men believe that FGM is women’s business and that their views on cultural obligation enabled the continuation of FGM. The women, therefore, perceived that men’s involvement as a crucial part of the solution to end this practice but it might be very challenging as men are not interested in taking part in such a movement. An example such as this was given:

*At the moment most of the trainings are for women. We need men to talk to men so we can engage them otherwise you cannot force them to sit in a class. You need to train more men to open up and talk about this issue with other men in the community and engage them at the same level as women. Men are still looking at it as a good thing.*

(W15)

Women described feeling empowered when they shared their stories and regarded these as an important resource for mutual support and to educate the community and challenge cultural beliefs about FGM. Women also mentioned that they feared being socially ostracised by their families and communities if they expressed dissenting views. This woman explained:

*…We need to create an environment where people talk about it. You know it is very hard to disclose such issues at community level, as it is a very private matter. I guess if we bring up stories and how women are suffering this would be effective to change this culture in the future. Imagine you’re living for someone else’s pleasure and you’re getting none.*

(W13)

Women considered government support as a cross-cutting issue linked to all future actions and approaches. Women used the word government to mean all high-level decisions, policy and funding at local, state and territory and federal levels. They wanted resources for improving the health of affected women, introducing FGM as a topic in the school curriculum and making meaningful linkages with communities. They believed such strategies would ultimately lead to the improvement of the health of women with FGM and society as a whole.

*They [policy makers] need to identify women with FGM as a priority at policy level and provide them with things they want. We want services which all women deserve …. We are in a developed country and we should have access to standard care from an experienced health provider.*

(W21)

Women also spoke of the need for mental health support and counselling services, both at facility and community level, for example:

*Make sure they [women affected with FGM] are OK, mentally and physically. Do the follow up afterwards. Education and individualised support not only for women who have undergone FGM but also to train staff and the community. It goes both ways.*

(W16)

Women pointed to cultural taboos that make it challenging to have open discussions about FGM with male members of the family. Several women made suggestions like this:

*Facilitating and funding community training such as workshops for men and women we can raise the awareness. It is also helpful to open the discussion around this issue. At the moment it is not culturally appropriate to even talk about it even in the family.*

(W20)

## 4. Discussion

This research identified the maternity care experiences of women affected by FGM and their views concerning the care they wished to receive in the future and how this might be achieved. Women in this study acknowledged that the maternity care they received had not always been at the level of quality that they desired or had expected. Women reported that being meaningfully involved in their care design and delivery was a crucial strategy for building trust and improving and validating the quality of maternity services. It has previously been shown that women who are well educated and have adequate information about FGM are more likely to have control over health care, access to shared decision making. Making an informed choice is key to respectful care for women with FGM [41] and they are less likely to perform FGM for their daughter [42]. While most women were motivated to be involved in their care, they struggled with poor communication and a lack of information tailored to their individual needs as reported elsewhere [43,44]. Women wanted to be cared for by skilled and culturally competent providers who treated them as ‘special’ but also as normal and equal to ‘other women’. This has been described by other research where they ensured equality by including Aboriginal and Torres Strait Islander midwives, who can interact holistically and provide culturally sensitive services [45]. Finally, women described the importance of having access to evidence-based models of care such as midwifery continuity of care and available services including, reconstructive surgery, management of trauma, emotional support, psychotherapy services and cultural support.

**Figure 2:**
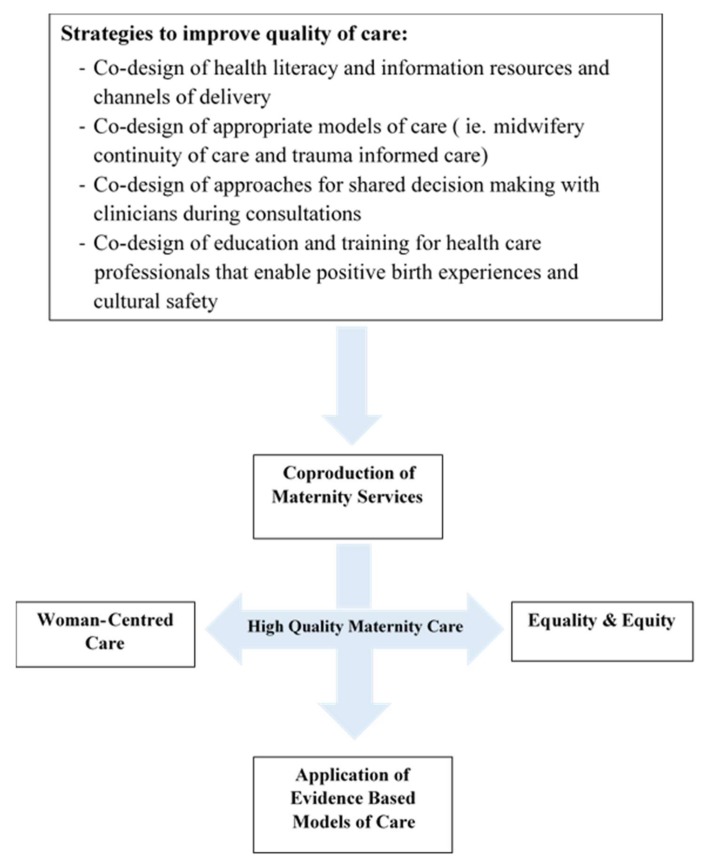
Conceptual model of quality improvement within maternity services for women with female genital mutilation (FGM).

A conceptual framework (Figure 2) was developed based on the findings of this research that highlights four priority approaches required to achieve quality care for women with FGM: co-production, woman-centred care, equity and equality and evidence-based models of care. These approaches are underpinned by four strategies that facilitate women’s engagement and include involvement in developing health information to being an equal partner in decision making and the co-design of maternity services. 

### 4.1. Co-Design of Health Literacy Interventions

Women regularly described the need for information that is tailored to their individual needs and noted that support services, such as counselling, were not always accessible due to language and cultural barriers. Some women stated that these services were not available or integrated into maternity care. This is similar to other studies in high-income countries that have found that women affected by FGM do not always receive or understand the information and resources they required or needed because of social isolation, stigma and a lack of health literacy [46]. When women have lower levels of health literacy, they are less prepared to engage and comply with their care regimes or protocols and as a result, do not receive optimum care [47]. Improving the health literacy of women with FGM may change the attitudes of women towards their own FGM and reduce the likelihood of their daughter‘s being circumcised [48].

Every woman should feel empowered to build her capacity and skills to use health information effectively and make an informed choice [49]. Many women in our study stated that they were not adequately engaged in their health care because of low levels of health literacy, inadequate information and unfamiliarity with their health rights. Again, these findings concur with other studies [50,51] and confirm that women’s participation in the process of health information design leads to more satisfying and positive experiences with enhanced health outcomes [52]. Health literacy programs that involve women designing and delivering programs not only build the capacity of women to facilitate the sharing of stories and experiences but also empowers women to support others in their community [53]. Such approaches are likely to be useful for women affected by FGM. 

### 4.2. Co-Design of Evidence-Based Models of Care

Most women in our study reported different types of FGM-related trauma, which affected their overall quality of life. Women expected health care providers to be responsive to their psychological, emotional and socio-cultural needs as found in other studies [54]. The central philosophy that underpins high-quality maternity care does not only involve a focus on physical health but also emotional well-being and includes quality of life issues [55]. Despite the emotional and mental consequences of FGM, most studies are focused on the physical aspects and implications [56,57]. Laio et al. [58] indicated that women affected by FGM are often silent about their emotional problems due to the stigma associated with FGM and have difficulty communicating with health providers. It is difficult for care providers to recognise or determine the level of psychological trauma that may be caused by FGM, but our study highlights the importance of these considerations. 

FGM related trauma is important to note because it can negatively impact on childbirth and sexual relationships highlighting the need for individualised trauma-informed interventions for such vulnerable women. FGM related mental health issues such as PTSD, negative body image and feelings of shame and stigma may also affect women’s health-seeking behaviour [59]. A trauma-informed model of care may be an approach to providing safe supportive care to women who have been affected by violence to reduce the consequences of trauma in their life [60]. 

Women should also be involved in the design of such trauma-informed services so that individual needs, views and experiences can be addressed in a collaborative way [61]. Efforts in the area of trauma-informed care currently focus on strengthening health provider’s knowledge and skills based on their interactions with consumers, rather than understanding a women’s experiences and needs [62,63]. Implementing participatory interventions, however, requires both the health system and community change [64]. Women need to be supported to become empowered to recognise their potential and utilise their capacity in the design and delivery of services [65]. Creating an environment of collaboration and mutual trust by engaging women and acknowledging their values and lived experiences may ensure that women’s needs are understood and their views and culture are taken into account in service design, thereby, improving the quality of culturally safe care.

Correa-Velez and Ryan [66] emphasise the need for specific models of maternity care for marginalised and high-risk women, such as women with FGM, that encompass continuity of care plus educational interventions and the delivery of mental health support. Our study indicates that continuity of care can lead to improved interpersonal communication and can boost women’s confidence, the collaboration between a woman and her provider and help facilitate women engagement in the process of care design and delivery [67]. Midwifery continuity of care enables health providers to consider the socio-cultural and emotional needs of marginalised women and, therefore, empowers women to achieve positive outcomes [68]. Such care models ensure the continuous assessment and evaluation of women’s experiences, opinions and views that can improve the quality of care for marginalised groups [69]. 

### 4.3. Co-Design Approaches to Shared Decision Making

The health system must offer women adequate support to enable them to be empowered to communicate, to ask for help and to question their care [60,70]. Patient participation in the process of service design and delivery is often missing as patients are perceived not to have adequate medical and clinical knowledge [71]. A review of the literature found that consumer involvement in the training of health providers ensures that the health system reflects their needs and desires in the design and delivery of services [72]. Collaborative partnerships have been found to have a positive impact on nursing practice by improving communication and shared decision making [73]. Another example from the field of mental health demonstrates the benefits of sharing the experiences and insights of patients through story-telling and using different aspects of personal experience in the development of a mental health assessment tool [74]. There is limited evidence in maternal health research and further research is needed to determine the best approach to engage women and evaluate the impact of their involvement in the co-design of education and training material, guidelines and health service processes.

### 4.4. Co-Design of Health Professional Education and Training

Women in our study described the need for health providers to receive special training on the cultural aspects of care for women from diverse backgrounds. This would help to address their need for a model of maternity care that integrates a woman’s cultural and individual values with excellent communication and referral paths to promote their well-being and safety as described in other research [75]. The involvement of women in teaching health professionals may be a useful strategy to increase the knowledge of clinicians. One study that investigated the outcomes of learning where consumers delivered classes found that nursing students improved their cultural knowledge and understanding of empathic care [76]. The involvement of mental health consumers in the education of nurses also showed improvements in nurses’ communication skills and decreased cultural barriers for consumers as well as reduced discrimination [77]. The integration of cultural safety in practice is challenging as it requires the involvement of service users in the co-design of such services and involving a vulnerable population requires a paradigm shift in power differences between service users and health professionals [51]. Future health services need to be co-produced with women to disrupt the inherent power imbalances. 

This study is one of the first of its kind in Australia to analyse this group of women’s views and experiences of their maternity care. The use of AI as the methodology was unique and enabled women to focus on their positive experiences and come up with solutions for future action and changes within the health system. The study has highlighted the voices of women providing important knowledge to improve the quality of maternity care for marginalised women. 

This study included only women who lived in Sydney, which is generally well resourced in terms of services for migrant populations. Therefore, the results may not be generalisable to the other states across Australia and suggested solutions and recommendations might be specific to the local context.

Sampling bias is a possible limitation. Potential women were recruited through chain referral sampling. Therefore, those who decided to participate in this study might be those who had more interest in this subject area, and this might have led the discussion either more positively or negatively. 

## 5. Conclusions

The engagement of individuals and communities is critical to the process of improving the quality of maternity health services and to address the socio-cultural needs of women affected by FGM. Empowering women and raising their awareness of their health care rights can help to engage women as active partners in the design and delivery of health information, models of care approach to shared decision making and health professional education and training which is based on their needs and context. 

Further research is needed to explore the replicability of the suggested framework at policy and practice levels. Research is required to establish the feasibility of the co-production of maternity services and how this improves the quality of care and equitable health outcomes for women affected by FGM.

## Figures and Tables

**Table 1:** Demographic information.

Study Code	Age	Age Underwent FGM	Country of Origin	Date of Last Birth in Australia	Education Level	First Language	Employment Status	# Children Born in Australia	# Live Birth	Years Lived in Australia
Astur	30–35	5–10	Somali	2012	Secondary	Somali	Employed	1	1	20–25
Bilan	30–35	5–10	Somali	2005	Primary	Somali	Housewife	5	5	20–25
Calaso	30–35	1–5	Somali	2013	Secondary	Somali	Employed	1	1	10–15
Bilqis	30–35	1–5	Somali	2010	Secondary	Somali	Employed	3	3	20–25
Indah	40–45	<1	Indonesia	2004	Tertiary	Indonesian	Employed	3	3	15–20
Aminata	40–45	10–15	Sierra Leone	2013	Tertiary	Creole Temne	Employed	2	3	10–15
Binta	25–30	5–10	Sierra Leone	2016	Tertiary	Temne	Employed	2	2	15–20
Arifa	30–35	1–5	Sudan	2013	Secondary	Arabic	Employed	3	4	10–15
Fiza	35–40	5–10	Sudan	2009	Tertiary	Arabic	Employed	2	2	15–20
Mariatu	25–30	15–20	Sierra Leone	2017	Secondary	Creole Temne	Housewife	2	2	5–10
Hiba	40–45	1–5	Sudan	2011	Secondary	Arabic	Housewife	3	5	10–15
Nadia	40–45	<1	Sudan	2006	Tertiary	Arabic	Employed	1	1	10–15
Rita	35–40	1–5	Sudan	2015	Tertiary	Arabic	Housewife	3	5	5–10
Yusra	35–40	5–10	Sudan	2017	Tertiary	Arabic	Housewife	4	5	5–10
Faduma	40–45	1–5	Somali	2009	Secondary	Somali	Housewife	5	5	15–20
Kia	35–40	1–5	Ethiopia	2011	Secondary	Arabic	Employed	3	3	15–20
Zara	25–30	5–10	Sudan	2016	Tertiary	Arabic	Housewife	2	2	10–15
Fatma	40–45	1–5	Sudan	2012	Secondary	Arabic	Housewife	2	5	10–15
Nour	30–35	5–10	Egypt	2016	Tertiary	Arabic	Employed	3	3	5–10
Gamal	35–40	1–5	Egypt	2015	Tertiary	Arabic	Employed	3	3	5–10
Asima	30–35	1–5	Sudan	2014	Tertiary	Arabic	Housewife	1	1	5–10
Harum	35–40	<1	Indonesia	2012	Tertiary	Bahasa	Housewife	2	3	5–10
Zaineb	40–45	1–5	Somali	2007	Primary	Somali	Housewife	1	1	10–15

## Supplementary Materials

The following are available online at https://www.mdpi.com/1660-4601/17/5/1491/s1.
