# Supplementary material for: Understanding the Experiences and Needs of Migrant Women Affected by Female Genital Mutilation Using Maternity Services in Australia
Source: Int J Environ Res Public Health. 2020 Feb 26;17(5):1491. doi: 10.3390/ijerph17051491 (PMC7084919; doi:10.3390/ijerph17051491)
Supplement: Supplementary file 1 [file ijerph-17-01491-s001.pdf]

Appendix 1: Field Guideline

Date and time:

Study ID:

Country of origin:

Years live in Australia:

Age:

Age Undergone FGM:

Number of Children:

Number of children born in Australia:

Date of latest birth in Australia:

Educational level (primary, secondary, higher education):

Job:

First language:

Research questions:

1. What do women affected by FGM perceive as quality maternity care?
2. How do women with FGM feel that their culture is acknowledged by maternity care services and how could this be improved from the perspective of cultural safety?
3. How do the relationships that women affected by FGM have with obstetricians, midwives and other health professionals during pregnancy and after birth impact on quality care?
4. How have women with FGM experienced maternity care decision-making and how do women think decision-making could be improved?
5. What expectations do women with FGM have of maternity health services in Australia and were they satisfied with the care they received?
6. How can women with FGM be more involved in the co-production of maternity care, services and information?

Note: we will use whichever term the women feel most comfortable with.

Questions for interviews and FGDs

The questions will be broad and will essentially address the 4 D model of appreciative inquiry – Discovery, Dream, Design and Develop. The 4D words in the model will not be used in the interviews but are provided here to illustrate the process.

Discovery (Individual interview)

I am interested in your experience with health care in Australia when you were pregnant and had your baby. Can you tell me how that was for you?

Depending on what the woman says, some trigger questions or additional points may include:

a) Was there anything that made it a good experience for you and your family?
b) What was the situation in that particular context that makes you satisfied?
c) Was there any times that were less good for you?
d) Did you feel you could decide what you wanted?
e) How were the doctors and midwives who cared for you? Did they do anything especially good?
f) How ere your cultural needs taken into consideration by the staff? (What was the core factor that effect your culture during this experience of healthcare and how?)
g) What else?

Dream (Individual interview)

a) If you had a baby again what would you like to happen that was different to what happened to you?
b) If your sister or daughter was to have a baby in the future, what would you like for her?
c) What would you do for women like yourself if you were a health provider in Australia?

Design (FGDs-Workshop)

a) What do you think needs to happen for your dream to be a reality?
b) What three wishes would you make to improve maternity for women affected by FGM?)
c) What is the most important thing that contributed to y our health during maternity care?
d) How can health services offer assistance in ways that women with FGM are most likely to accept help and benefit from it?

Develop (FGDs-Workshop)

a) What can we do together to contribute to your dream?
b) In what way would you are your family or community like to be involved?
